# Machine and Deep Learning towards COVID-19 Diagnosis and Treatment: Survey, Challenges, and Future Directions

**DOI:** 10.3390/ijerph18031117

**Published:** 2021-01-27

**Authors:** Tarik Alafif, Abdul Muneeim Tehame, Saleh Bajaba, Ahmed Barnawi, Saad Zia

**Affiliations:** 1 Computer Science Department, Jamoum University College, Umm Al-Qura University, Jamoum 25375, Saudi Arabia; 2 Department of Software Engineering, Sir Syed University of Engineering and Technology, Karachi 75300, Pakistan; muneeim301@gmail.com; 3 Business Administration Department, King Abdulaziz University, Jeddah 21589, Saudi Arabia; sbajaba@kau.edu.sa; 4 Faculty of Computing and Information Technology, King Abdulaziz University, Jeddah 21589, Saudi Arabia; ambarnawi@kau.edu.sa; 5 IT Department, Jeddah Cable Company, Jeddah 31248, Saudi Arabia; saadzia_00@hotmail.com

**Keywords:** COVID-19, diagnosis, treatment, artificial intelligence, machine learning, deep learning

## Abstract

With many successful stories, machine learning (ML) and deep learning (DL) have been widely used in our everyday lives in a number of ways. They have also been instrumental in tackling the outbreak of Coronavirus (COVID-19), which has been happening around the world. The SARS-CoV-2 virus-induced COVID-19 epidemic has spread rapidly across the world, leading to international outbreaks. The COVID-19 fight to curb the spread of the disease involves most states, companies, and scientific research institutions. In this research, we look at the Artificial Intelligence (AI)-based ML and DL methods for COVID-19 diagnosis and treatment. Furthermore, in the battle against COVID-19, we summarize the AI-based ML and DL methods and the available datasets, tools, and performance. This survey offers a detailed overview of the existing state-of-the-art methodologies for ML and DL researchers and the wider health community with descriptions of how ML and DL and data can improve the status of COVID-19, and more studies in order to avoid the outbreak of COVID-19. Details of challenges and future directions are also provided.

## 1. Introduction

Severe Acute Respiratory Syndrome Corona-Virus 2 (SARS-CoV-2) is currently a considerable infectious worldwide disease. Corona-virus 2019 (COVID-19), which was caused by a virus named SARS-CoV-2, was very first apprised in Wuhan, China in December 2019 and later in many parts all over the world; on 3 January 2020, the World Health Organization declared that COVID-19 is a Public Health Emergency of International Concern (PHEIC), and confirmed it as an epidemic on 11 March 2020 [1]. This disease has been accounted for in 216 countries and regions all around from 16 May 2020. The disease has spread and prompted momentous side effects, with 86,159,886 cases of confirmed coronavirus and 1,861,764 deaths on 5 January 2021.

The health industry is eagerly looking for new technologies and techniques to track and control the growth of coronavirus epidemic in this international health crisis. One of the greatest uses global technology right now is Artificial Intelligence (AI), which can track the speed and detect the growth rate of the corona virus, and identify the risk and severity of Corona virus patients. AI can also anticipate the possibility of death by adequately analysing previous patient data. Artificial intelligence can assist us in battling the virus by testing individuals, medical assistance, data and information, and recommendations regarding disease control.

In order to solve complex problems in our lives, AI is a broad umbrella that consists of many sub-areas. These sub-areas include learning, preparation, thinking, representation of information, and searching. Machine Learning (ML) and Deep Learning (DL) are a subset of AI areas that consist of several algorithms that provide intelligent models to identify or cluster particular tasks.

ML is a subset of AI that consists in the algorithmic modeling culture of statistical models [2], and only needs a small amount of knowledge to learn how to solve problems. Logistic Regression (LR), Decision Tree (DT), Random Forest (RF), K-nearest Neighbor (KNN), Adaboost, K-means clustering (KC), Density clustering (DC), Hidden Markov Models (HMM), Support vector machine (SVM), Naive Bayes (NB), Restricted Boltzmann Machines (RBM), and Artificial Neural Network (ANN), such as Recurrent Neural Networks (RNN), including Long-short-term-memory (LSTM), Autoencoder (AE), and Generative Adversarial Network (GAN), are ML techniques.

DL, on the other hand, is a subset of ML that focuses on building deep structural NN models that learn from data using algorithms of feedforward and backprobagation. After ML, the DL emerged and outperformed it in the last two decades in several activities. Nevertheless, it takes a huge amount of data to understand. Exceptional cases of DL, where large-scale data are not needed to train, have been transfer learning and generative models. DL algorithms usually involve Deep Belief Networks (DBN), Deep Neural Network (DNN), and Deep Convolutional Neural Networks (Deep CNN).

Appreciatively, research works in industry, medical, technological, and military sectors have victoriously introduced advanced AI-based ML and DL methods in the COVID-19 war within a short period after the outbreak of COVID-19 and attained substantial progress. For example, across medical image analysis, ML and DL help COVID-19 diagnosis as well as provide non-invasive detection measures to avoid medical personnel from contracting pathogens and, for further treatment, the patient’s severity score is also given. In virology studies, ML and DL are used to examine SARS-CoV-2 protein-related genetics and predict novel combinations that can be used for drug production and vaccination. In addition, on a large-scale, COVID-19 case data and social media data, AI intelligent models that are based on ML and DL learn to construct disease transmission models that accurately predict outbreaks, transmission path, transmission list, and effects. ML and DL are also vastly used in epidemic protection and public monitoring, such as security check-ups in airports, patient tracking, and epidemic detection.

**Figure 1:**
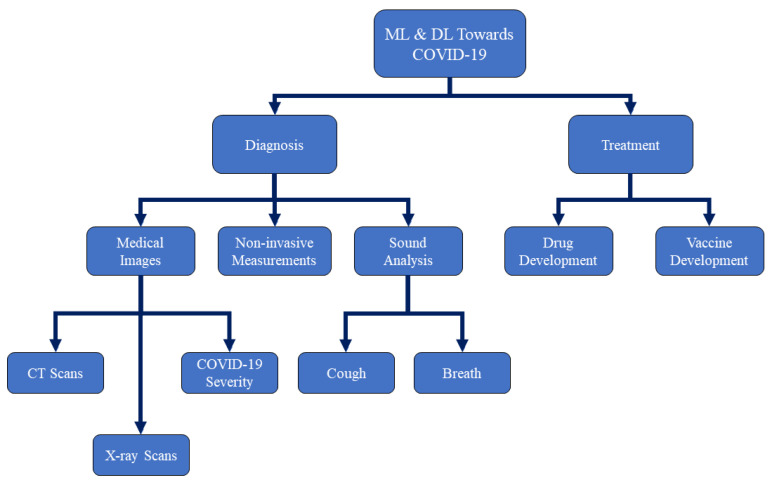
A taxonomy of our survey on Machine Learning (ML) and Deep Learning (DL) research works towards Corona-virus 2019 (COVID-19) diagnosis and treatment.

In this survey, we introduce the main scope of AI focusing on ML and DL towards COVID-19 research incorporates the sides of disease diagnosis and drug and vaccine developments. Note that, due to the fast evolution of the COVID-19 epidemic, we have quoted many published research works before a thorough investigation, where these works should actually be surveyed for their precision and quality in peer review. Figure 1 shows a taxonomy of our survey on ML and DL research works towards COVID-19 diagnosis and treatment.

The remainder of this paper is organized, as follows. In Section 2, we provide details on the current AI-based ML and DL approaches for classifying COVID-19 from Chest CT and X-ray scans in addition to non-invasive COVID-19 measurements. We also show the COVID-19 severity detection while using the DL-based methods. In Section 3, we provide details on the current AI-based ML and DL approaches for drug and vaccines development. In Section 4, challenges and future work directions while using AI-based ML and DL approaches for tackling the problem of COVID-19 are provided in details. Finally, Section 5 provides our final comments.

## 2. Medical Image Inception Using AI-Based ML and DL for the Detection of COVID-19

Nationally and internationally, the Coronavirus (COVID-19) outbreak is increasing. In the universal battle against COVID-19, for example, medical imaging, X-ray, and computed tomography (CT) play a key role, and the latest AI developments tend to improve the capacity of imaging tools and facilitate healthcare personnel.

Medical imaging research is commonly used for the identification of COVID-19 by clinicians. Chest X-ray and lung CT image samples are mostly used in COVID-19 clinical imaging trials. AI innovation plays a significant role in medical imaging testing. It has produced enormous results in image identification, organ recognition, geographic infection classification, and disease classification. It not only decreases the picture diagnostic time of the radiologist, but it also increases the accuracy and execution of the diagnosis. AI can enhance work performance through correct diagnostic precision in X-ray and CT imaging, which makes it easier to test, as follows. The computer-aided networks also assist radiologists in making clinical decisions, i.e., for the identification, monitoring, and prognosis of diseases. We will address the innovations of AI techniques to chest X-ray and CT imaging in depth.

### 2.1. Chest CT Image Detection

A valued feature of the assessment of patients with doubtful SARS-CoV-2 infection is the chest CT picture. There is a growing research on the role of COVID-19 imaging for treatment and diagnosis. The infection triggers a huge spectrum of CT scan imaging discoveries, most commonly ground-glass opacities and lung periphery consolidations. Chest CT sensitivity to diagnose COVID-19 has been found to be significantly higher and it can occur prior to a positive viral lab test. Therefore, hospitals with a large quantities of admissions use CT for the fast emergency of patients with conceivable COVID-19 disease in epidemic territories, where the basic healthcare system is under pressure. Chest CT plays a vital role in the estimation of COVID-19 patients with severe and compound respiratory symptoms. Based on scans, it is possible to determine how badly the lungs are compromised and how the illness of the individual progresses, which is effective in making medical decisions.There is a growing understanding of the sudden incidence of lung defects that are induced by COVID-19 in CT scans that were conducted for many other clinical indications, such as abdominal CT scans for bowel disorders or patients without respiratory symptoms [3]. In this pandemic, by reducing the strain on clinicians, the evaluation of AI may become the most significant factor. Although it can take up to 15 min. to manually interpret a CT scan, AI can analyse the images in 10 s. [3]. Therefore, advanced image processing with artificial neural network has the possibility to significantly improve the function of CT in COVID-19 detection by allowing a large proportion of patients to identify disease easily and rapidly with accuracy. The continuation of AI-based CT imaging tests usually involves the following steps: regional division of the Region of Interest (ROI), removal of pulmonary tissue, identification of regional infection, and classification of COVID-19. A basic basis for analysing AI-based imagery is the recognition of lung organs and ROIs. ROI has been demonstrated for further testing and analysis in CT imaging in lungs, lung lobes, bronchopulmonary segments, and regions with infection or ulcers. For CT image classification, different types of DL networks, e.g., U-Net, V-Net, and VB-Net, VNET-IR-RPN, had been used. From an overall of 905 patients assessed with real-time RT-PCR assay and next-generation RT-PCR, 419, approximately (46.3%), were confirmed by an AI device with SARS-CoV-2. The AI method consists of deep CNN for the primary CT scan to evaluate the image characteristics and attributes of individuals with SARS-CoV-2. Subsequently, according to clinical knowledge, SVM, RF, and MLP classifiers were used in order to identify SARS-CoV-2 patients. To foresee COVID-19 status, the AI system operates on radiological data and medical factors. The deep CNN-based AI system obtained an AUC of 92% and it had a comparable sensitivity relative to the senior thoracic radiologist in the experimental set of 279 patients. Furthermore, the Artificial Intelligence (AI) system enhanced the detection of patients, who aimed for RT-PCR detected COVID-19 who submitted standard CT scans, which correctly classified 17 out of 25 patients (68%), and all of these patients were graded by radiologists as COVID-19 negative [4]. The training dataset also included 25 COVID-19 positive cases with a chest CT marked as negative by the two reading radiologists during display. The CNN-based model categorized 13 out of 25, about (52%) of images, as positive for COVID-19. The clinical model categorized 16 out of 25, about (64%) of images, as positive for disease, and the joint model categorized 17 out of 25 (68%) as positive for disease, while the senior radiologist and their fellows classified 0 out of 25 (0%) of these images as being positive for disease [4].

In an attempt to discover the answer that could rapidly decompose images from deep learning and recognize COVID-19 features, researchers are trying to develop several AI resources. A research team led by Bo Xu of the Tianjin Medical University Cancer Institute and Hospital involved CT scans of 180 patients that were confirmed to have severe viral pneumonia even before epidemic of COVID-19 and 79 patients with certified COVID-19 to establish an AI method to classify COVID-19 [5]. They gave pictures of patients at random to train and test a deep CNN-based algorithm. In the findings that were released in medRxiv [6], the researchers stated that their model detected COVID-19 with 89.5% accuracy from CT images. An accuracy of approximately 55% was reported by two radiologists who analysed the images. The team confirms the findings indicate that from a CT scan, AI can provide an exact analysis. Another algorithm, named as RADLogics algorithm [7], managed to detect and assist in initiating a COVID-19 patient’s improvement.

Two studies, published in [4,8], advance this thought by utilising DL trained on CT scans as a fast symptomatic instrument to search for COVID-19 infection in sufferers who were admitted to the hospitals and required medical image processing. In [8], researchers at Macau University of Science and Technology applied 532,000 CT images from 3777 patients in China to train and test their AI-based models, concentrating on the tell-tale lesions observed in COVID-19 patient lungs. The AI model successfully diagnosed coronavirus-induced pneumonia no less 85% of the time when it was used in a database of 417 patients in four separate groups in a pilot study across many Chinese hospitals.

There seems to be a big problem in distinguishing whether the signs are COVID-19 and pneumonia detected in CT images by radiologists. A company, VIDA Diagnostics [9], has developed a LungPrint device that utilises AI to analyse CT scans to accurately identify respiratory disorders, including COVID-19 signs and symptoms. In [10] NIH and NVIDIA researchers attempted to create a DL technique to detect COVID-19 using chest CT images utilising datasets from four hospitals across China, Italy and Japan. In total, in this study, researchers used 2724 samples from 2619 patients, including two models (i.e., Full 3D, Hybrid 3D) that were used in series to establish the final prediction model for COVID-19. These two models work. The first model implemented a fixed input size (full 3D) of the entire lung area. At fixed image resolutions (hybrid 3D), the second model used an average score for a few regions in each lung. The hybrid three-dimensional (3D) model achieved 92.4% validation accuracy when detecting COVID-19 as well as other pneumonia, whereas the full 3D model achieved 91.7% accuracy.

In order to retrieve ROIs from each CT image and acquire a training curve for suspected lesions, Chen et al. [11] constructed a U-Net++ deep learning structure. 46,096 anonymous images were collected and processed for model creation and validation from 106 patients already admitted, including 51 laboratory patients who had reported COVID-19 pneumonia and 55 other disease control patients at Renmin Hospital of Wuhan University in China. On 5 February 2020, twenty-seven consecutive patients experiencing CT scanning were grouped at Renmin Hospital of Wuhan University in order to estimate the effectiveness of radiologists as compared to the 2019-CoV pneumonia model. The U-Net++ model achieved an overall of sensitivity of 100%, specificity of 93.55%, accuracy of 95.24% while using retrospective dataset. Huang et al. [12] used the AI-based InferReadTM CT pneumonia method to accurately assess improvements in the lung burden of COVID-19 patients. Three modules are incorporated into the tool: pulmonary and lobe extraction, classification of pneumonia, and measurable analysis. The CT image characteristics for COVID-19 pneumonia are divided into four modalities: mild, moderate, severe, and critical. A professional deep learning software automatically calculated the level of CT lung natural action of the overall lung and five lobes, and compared CT scans over follow-up. A total of 126 COVID-19 patients, including six mild, 94 moderate, 20 severe, and six critical cases, were assessed. The rate of CT-based natural action was entirely diverse among the initial clinical groups, progressing gradually from mild to severe (all *p* < 1%).

71 CT scans from 52 COVID-19 approved patients in five hospitals were obtained by Qi et al. [13]. They used the Pyradiomics methodology to excerpt 1218 traits from each CT image. The models of CT radiomics focused on LR and RF algorithims. They were built on pneumonia lesion extracts during training and interactions. At the lung lobe and the patient level, predictability efficacy was also evaluated in the experimental database. The types of CT Radiomics are focused on six second order. They were successful in distinguishing short-term and long-term stays in patients with SARS-CoV-2-related pneumonia, with 97% AUC and 92% LR and RF, respectively. The LR model showed 100% and 89% sensitivity and specificity, while the RF model showed 75% and 100% similar sensitivity and specificity. The short-term hospital stay is less than 10 days, while the long-term hospital stay is more than 10 days.

### 2.2. Chest X-ray Image Detection

**Figure 2:**
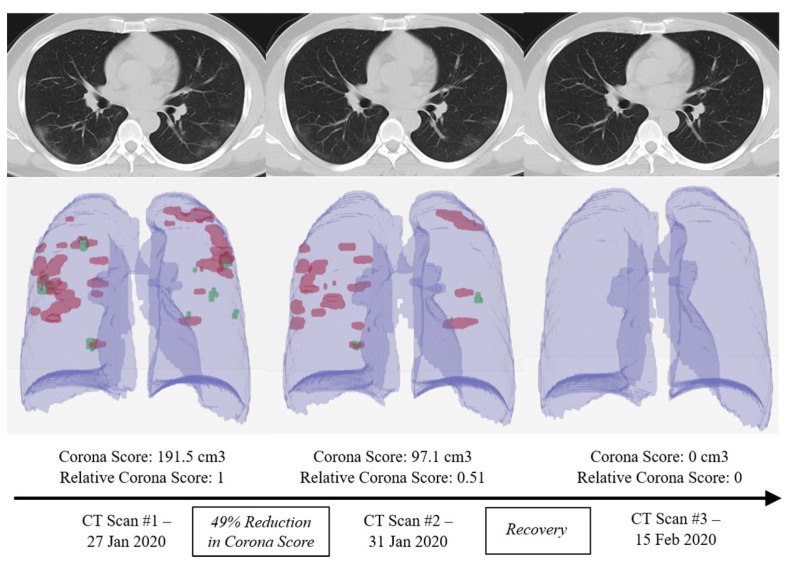
Using different computed tomography (CT) scans from a a COVID-19 patient, the RADLogics algorithm measures the recovery amount using a specific score [16].

Chest X-rays have been proposed as a highly helpful method for evaluating and testing COVID-19 patients. Figure 2 shows representative architectures of DL-based CT image classification and COVID-19 examination. When compared with CT images, chest X-ray (CXR) images are simpler to acquire in clinical radiology examinations. There are many available studies [14,15] that operates on chest X-ray (CXR) images for corona virus detection. For the most part, the CXR image testing factor that is based on AI strategies involves measures, such as data correction, model training, and segmentation of COVID-19. There are several methodologies of deep learning (such as CNN, nCOVnet, and U-Net++) that are used to find better and fast detection in the detection of COVID-19 on X-ray images.

In medical centers and hospitals, X-ray devices deliver less costly and quicker outcomes from scanning different human organs. The interpretation of numerous X-ray images is typically performed by radiologists manually. Radiologists are only equipped to detect 69% of X-ray COVID-19 cases [17]. Pre-trained models made it much easier and quicker to detect COVID-19. In [14], a dataset of reported positive COVID-19, typical bacterial pneumonia, and stable (no infection) cases was used. In this survey, a total of 1428 X-ray scans were applied. The authors used the pre-trained model of VGG-16 in order to model the role of division and perform the categorization. In two and three production class cases, the examiner obtained 96% and 92.5% accuracy, respectively. Based on the obtained results, X-ray images can be accessed by the medical community as a possible symptomatic method for immediate and faster COVID-19 identification to supplement the current diagnostic and symptomatic approaches. Several more creative algorithms are used for better results by CXR images towards the war with SARS-nCOV-2. By assessing essential characteristics from chest X-ray scans, Basu and Mitra [15] introduced a domain transfer learning for detecting COVID-19. The definition of Gradient Class Activation Map (Grad-CAM) is also used on the collection of 20,000 Chest X-rays to get an account of the COVID-19 detection. In order to obtain an overview of the viability of using chest X-rays tuned for classification between four groups, i.e., normal, other disease, pneumonia, and COVID-19 data, were used to diagnose the disease using a five-fold cross validation. With 100% of the COVID-19 and normal cases being accurately characterised in each validation fold, the overall accuracy was computed as 95.3%. A misdiagnosis in both pneumonia and other disease stages has occurred.

Students were also involved in the production of ML algorithm to identify novel COVID-19 patients. With the assistance of AI, Cranfield University students developed computer models that can diagnose COVID-19 in CXR images [18,19]. ML and DL techniques were used in the proposed models in order to obtain characteristics and identify CXR images. It can discern data that would not generally be apparent to the naked human eye and aid with COVID-19 detection.

**Figure 3:**
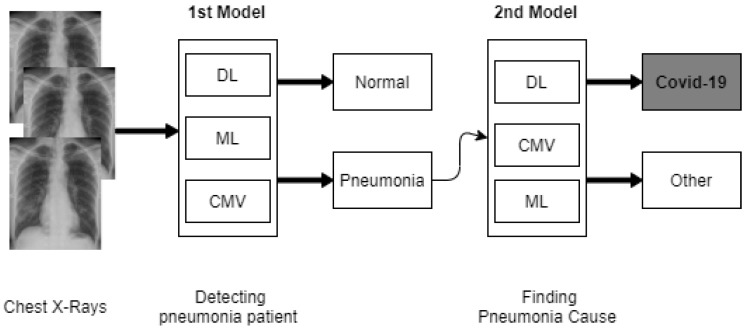
Our representation of the proposed computation intelligent models used at Cranfield University [18,19].

The first model is to examine abnormalities in an X-ray, distinguishing patients with normal and pneumonia. The second model further operates on certain patients with pneumonia in order to determine whether the COVID-19 virus causes pneumonia [18,19]. Figure 3 shows a high-level representation of the intelligent computational models that were developed at Cranfield University.

In the meantime, previous researches at King’s College London, Massachusetts General Hospital and health tech company Zoe have begun studies of a fully AI-based identification that aims to predict COVID-19 pathogens by means of assessing the symptoms with the implications of traditional COVID-19 tests [20]. With the support of Chest X-ray scans for COVID-19 diagnosis, another model [21] was proposed in order to provide an end-to-end structure without even using an extracting features tool. This model is built on the foundations of Darknet-19 (form based on a real-time object detection method, called YOLO), named DarkCovidnet, [21], which is already documented. The achieved 85.35%, 92.18%, and 87.37% of sensitivity, specificity, and F1-score values, respectively, while using the same model.

Most research studies are conducted using various methods in order to resolve COVID-19. Mangal et al. [22] proposed a COVID-19 AI-based Detector (CovidAID). CovidAID is a deep neural network model that was developed on the publicly available covid-chestxray-dataset dataset to treat patients for proper examination. With 100% sensitivity, they achieved 90.5% accuracy. In order to demonstrate COVID-19 positive X-rays from other negative ones, a deep learning-based CNN model, called Truncated Inception Net, was proposed in [23]; six distinctive dataset forms were carried out by means of the types of X-rays: COVID-19 positive, pneumonia positive, tuberculosis positive, and normal cases. In detecting COVID-19 positive cases from total Pneumonia and healthy patients, their model obtained 99.96% accuracy (AUC of 100%). Comparably, in classifying COVID-19 positive patients from other types of X-ray scans, it obtained an accuracy of 99.92% (AUC of 99%). They proved the feasibility of employing the proposed Truncated Inception Net as a screening method, according to [23], and outperformed all of the existing tools. Research on COVID-19 prediction in X-ray images using transfer learning was published by Minaee et al. [24]. Four common pre-trained deep convolution neural networks (CNNs), which are ResNet18, ResNet50, SqueezeNet, and DenseNet-161, were compared with their predictions They use COVID-19 and non-COVID datasets to instruct these four models, including 14 subclasses containing normal ChexPert dataset images. The models resulted in an average specificity rate of ∼90% with a sensitivity range of 97.5%. This highly supports the possibility that CXR imaging will distinguish COVID-19 from other infections and normal lung conditions. A COVID-19 assessment DT classifier from Chest X-ray Imaging was applied by [25]. It included three binary DTs, each of which was trained by a deep learning model that was built on the PyTorch system with a CNN. The Chest X-ray images were graded as normal or abnormal by the first DT. The second tree differentiated the abnormal X-ray images containing tuberculosis symptoms, while the third tree distinguished the equivalent second for COVID-19. The accuracy of the first DT is 98% and the second DT is 80%, while the third DT’s average accuracy is 95%. They claimed that the suggested deep learning-based DT classifier can be applied before RT-PCR outcomes are available in pre-screening instances in order to perform triage and fast-track conclusion making. GAN (Generative Adversarial Network) was introduced in a study [26] with deep transfer learning for COVID-19 identification in chest X-ray scans with a shortage of chest X-ray images dataset. 307 images for four different class groups, i.e., COVID-19, normal, bacterial pneumonia, and virus pneumonia were collected. Three AlexNet, GoogLeNet, and ResNet18 deep transfer models were chosen for operation. In this analysis, three case scenarios were tested: the first scenario consists of four dataset classes, while the second scenario consists of three classes, and the third scenario consists of only two classes. It was a must to have the COVID-19 class in all scenarios. In the first scenario, as it attained 80.6% in testing accuracy, GoogLeNet was chosen to be the essential deep transfer model. With the use of Alexnet, the second scenario obtained a testing accuracy of 85.62%. GoogLeNet was selected as a fundamental deep transfer learning model in the third scenario (which involves two classes, i.e., COVID-19 and normal), which gave an ideal 100% accuracy in testing and 99.9% accuracy in validation.

Especially in the low-resource X-ray settings may play a significant function in COVID-19 triage. 24,678 X-ray images were used in order to classify COVID-19 [27]. A deep learning-based AI framework CAD4COVID-Xray was trained. A lung segmentation with U-net and a CNN was implemented. 454 images were analysed from a dataset (223 patients tested positive for COVID-19, the remaining 231 tested negative for COVID-19). Chest X-ray images were specifically labelled as COVID-19 pneumonia with an AUC of 81% by the AI system CAD4COVID-XRay. According to author [27], as part of a process of looking at symptomatic issues, the system can be useful, especially in low-resource settings, where diagnostic equipment is not accessible. The Table 1 provides a summary of the best score AI-based ML and DL methods for diagnosing COVID-19 while using radiology images.

### 2.3. COVID-19 Severity Classification Using Chest X-ray with Deep Learning

**Figure 4:**
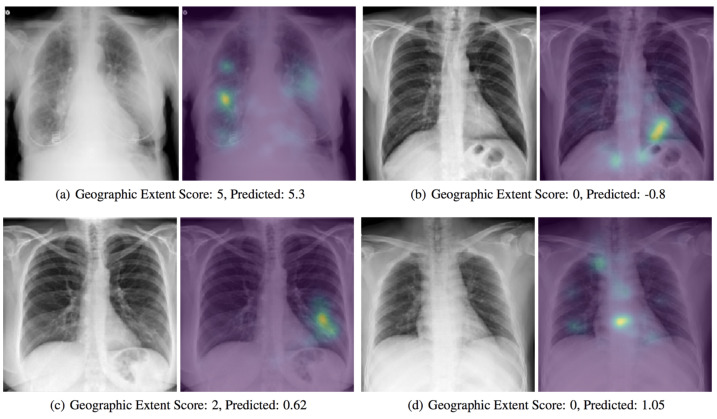
A presentation of a predicted severity scores for COVID-19 chest X-ray scans while using the DenseNet model [48].

With the support of artificial intelligence, chest X-rays enable us to understand more, particularly by using ML and DL techniques. Chest X-rays (CXRs) offer a non-invasive method for monitoring disease progression. For front-chest X-ray pictures, a severity score predictor model of COVID-19 pneumonia is being studied in [48]. Expansions of lung involvement and light intensity are also included in the CXR image database. A pre-trained neural network model (i.e., DenseNet) in large chest X-ray sets (non-COVID-19) is used in order to create features of COVID-19 images to predict this activity. 94 images of COVID-19 certified patients go to studying the severity of COVID-19 prediction while using DL, as shown in Figure 4. A score-based methodology is used, which includes two forms of scores. Table 2 provides examples of disease severity stages: the level of lung participation and the degree of ambiguity are shown at a time in a single X-ray image.

For each lung, the level of lung involvement by ground glass ambiguity and degree of unification were rated. The overall degree and ambiguity score on both the right and left lungs ranged from 0 to 8 and 0 to 6, respectively. The severity of a disease that are used for the increase or decrease of care, as well as tracking the effectiveness of patient treatment, especially in the ICU, can be found on the basis of the score.

Mobile app development also utilises AI to evaluate the severity of COVID-19 in the game. In [49,50], a mobile app was created by researchers from NYU College of Dentistry that used a dataset of 160 images of reported patients with COVID-19 from China. While using the mobile app, the numerous bio-markers that were contained in the blood will diagnose COVID-19 severity levels from level 0 (mild) to 100 (extreme).

Ridley [51], on the other hand, developed a special form of deep-learning algorithm, called the Convolutionary Siamese Neural Network (CSNN) to generate a score of COVID-19 patient pulmonary X-ray severity (PXS) and related well with radiologist evaluations, and could also help to predict whether a patient will require intubation or not before dying. The algorithm was carried out with two kinds of internal and external datasets. Internal research was conducted on a dataset of 154 COVID-19 admission chest X-rays, of which 92 had additional chest X-ray follow-up and were used for longitudinal study. In a community hospital, Newton-Wellesley Hospital in Newton, MA in the United States, external testing was performed on 113 consecutive admission chest X-rays from COVID-19 cases. The researchers found that the median PXS score was higher in intubated or deceased patients (PXS score = 7.9) as compared to those who had no intubation (PXS score = 3.2) on all of the test sets. The difference (*p* < 0.001) was statistically important.

Qi et al. [13] obtained 71 CT scans from 52 COVID-19 patients that were registered in five hospitals. They applied the methodology of pyradiomics to extract 1218 attributes from all the CT images. Logistic regression (LR) and random forest (RF) based CT radiomics models were built on pneumonia lesion extracts in testing and interactions. Predictability efficiency was also evaluated in the experimental database at the lung lobe and at the patient level. The types of CT in radiology are based on six-second order that have been functional in distinguishing short-term and long-term retention in patients with SARS-CoV-2-related pneumonia, with 97% and 92% AUC by LR and RF, respectively, in the testing phase. The LR model resulted in 100% of sensitivity and 89% of specificity, while the RF model resulted in 75% and 100% in sensitivity and specificity in the test results. Hospital stay for short-term is less or equal than 10 days, while hospital stay for long-term is larger than 10 days.

### 2.4. Observing COVID-19 Through AI-Based Cough Sound Analysis

Coughing is a symptom of more than 30 medical conditions that are not COVID-19. This makes COVID-19 infection identification by coughing alone a big challenge for different problems. Physicians also use sound signals that are made by human bodies. Examples of these sounds are moaning, breathing, heartbeat, digestion, and vibrating sounds. They are considered as indicators of diagnosis. Up to this point, at scheduled visits, such signals were usually collected via manual auscultation. Other research is being conducted for cardiovascular and respiratory studies while using digital technologies to capture body sounds (e.g., from digital stethoscopes), which can be used for automatic COVID-19 analysis. Recent research has begun to investigate how respiratory sounds (e.g., cough, breathing, and voice) that are recorded in hospital by devices from patients that tested positive for COVID-19 differ from healthy people’s sounds.

An analysis of identification of COVID-19 based on coughs that were collected via phone app is reported in [52], using a cohort of 48 COVID-19, 102 bronchitis, 131 pertussis, and 76 normal cough sounds to learn and evaluate this diagnostic method. Equation (1) is used in [52] to transform the collected cough data into Mel scale *m* for data pre-processing:

(1) $$m=2595\times log_{10}\left(1+\frac{f}{700}\right)$$

where *m* is a pitch scale that is measured by listeners to be equal in distance from each other [53]. Another mobile app, named kAs, was built in [54], by Zensark Technologies (Hyderabad, Telangana, India) while using machine learning to assess patient respiratory health and disease-specific cough signatures. The app provides 15 questions regarding the subject and their cough sound into the mobile app to transmit the cough sounds to Swaasa, the company’s AI platform, where on the basis of the answers to the questionnaire and the coughing tone, it goes to an audiometric examination and generates a COVID-19 Risk Score. The app produces a rating on a scale of 1 to 10, where 10 is the highest risk level. It may take days or weeks to monitor person’s risk score.

Schuller et al. explored the effective use of Computer Audition (CA) and AI in the study of cough sounds in COVID-19 patients [55]. They first tested the capacity of CA to detect speech and cough automatically in different conditions, such as breathing, dry and wet cough, or sneezing, flu-like speech, eating habits, sleepiness, or pain. They subsequently recommended using the CA technology in the diagnosis and cure for COVID-19 patients. However, there is no documentation on the use of this technology in COVID-19 research, due to the absence of usable information and annotated data. Wang et al. [56] studied the respiratory tract of patients with COVID-19 and other usual cold and flu respiratory tract patients. In addition, for the diagnosis of COVID-19, they suggested a model of respiratory simulation (called BI-AT-GRU). The BI-AT-GRU model involves the bidirectional bids and attention of the GRU neural network, and it can differentiate between six forms of clinical respiratory types, such as Eupnea, Tachypnea, Bradypnea, Biots, Cheyne-Stokes, and Central-Apnea. Sharma et al. [57] intended to create the laboratory diagnostic techniques of COVID-19 for sputum testing. In roder to test biomarkers in acoustics, the project, called Coswara, used cough, breath, and speech sounds. Data were collected for nine distinct vowel sounds. Some other features were also collected. The nine voices carry on varying patterns of body-breathing. Visual and temporary spectral features have been extracted from audio files. Tasks for classification and data curation are under operation.

**Figure 5:**
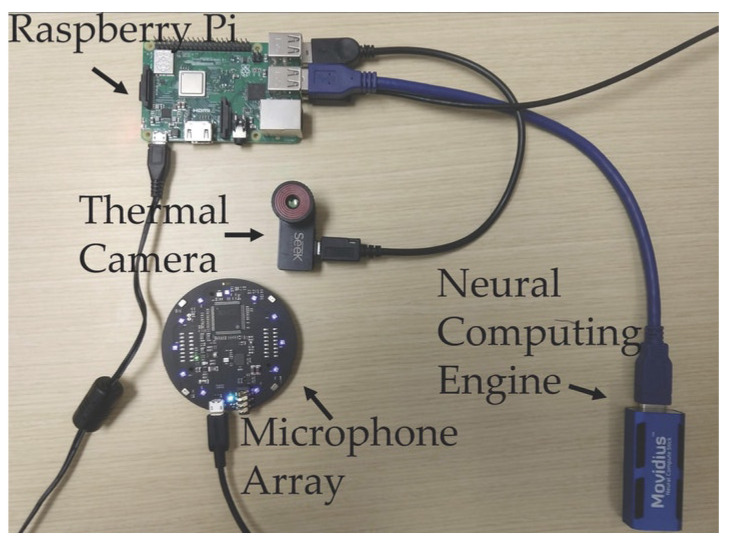
The FluSense device houses these components [54,58].

A portable device, called FluSense [54,58], was developed by the University of Massachusetts Amherst. Figure 5 shows the components of the FluSense machine that was operated by an AI-based neural network that can real-time identify cough and crowd size and directly evaluate and collect data for flu-like diseases, such as COVID-19. A microphone array and a thermal camera configuration and neural computing engine are used by the FluSense to actively and constantly render speech and cough signals as well as real-time adjustments in the crowd density on the edge. These sources of knowledge help to determine the time of campaigns for vaccination for the flu, possible travel restrictions, delivery of drugs, and more [59]. FluSense developers claim that the new edge computing platform, which is believed to be used in medical centers, will extend the health monitoring instruments that are used to predict influenza and other viruses, including the epidemics of COVID-19.

In [58], it has shown that FluSense accurately predicted the daily number of patient while using a Pearson Correlation Coefficient of 95%. Respiratory diseases or additional health data were both not considered by the FluSense platform. Information on cough is important and necessary, but it is not appropriate for all respiratory infections to be used. They gather data from a number of sources. An online website devoted to spreading cough sounds from COVID-19 patients, Ravelo [60], launched a cough AI-based system against COVID-19. The Bill and Melinda Gates Foundation is supportive of this initiative. The mechanism is plain. A individual downloads a sputum recording and it is requested to provide information, such as symptoms, other diseases, gender, and geographic location, in order to decide whether the person is from a specific area has a COVID-19 infection or not. The procedure also asks individuals for a photo of the results of their COVID-19 test. They will create an ML or DL-based algorithm once they have enough data and check whether the cough sounds that are associated with COVID-19 infection can be accurately determined. Iqbal et al. [61] addressed an anonymous structure that applies the role of the mobile app recognition to obtain and evaluate suspicious cough sounds of individuals in order to assess whether a person is healthy or suffering from respiratory disease.

A cross-sectional system between vocal cords (coughing and breathing) was created by researchers at the University of Cambridge [62] in order to recognise safe and unhealthy individuals. Speech sounds are applied to differentiate between COVID-19, normal persons, and asthma. The three binary separation functions are structured, as follows:

- Separating positive users of COVID-19 from negative users.
- Separate COVID-19 cough users from healthy cough users.
- Separate COVID-19 cough users from asthma users who have declared coughing.

In the community driven data collection, more than 7000 unique users (approximately 10 K samples) participated, from which more than 200 registered positive COVID-19. The typical methods of audio enhancement have been used to maximise a data set’s sample size. For the classification task, three classifications were used, namely, LR, Gradient Boosting Trees (GBT), and SVM. In order to compare the efficiency, the analysis used the combined curve area under the curve (AUC). In all three binary split trades, more than 70 percent of AUCs are registered. In order to distinguish, the researchers used respiratory tests and found that the AUC was around 60%. However, because of the high number of characteristics, the AUC progresses to around 80% per operation when coughing and the respiratory inputs are merged into phases. Table 3 provides a description of AI-based ML and DL approaches for speech and audio analysis regarding health problems with COVID-19.

### 2.5. COVID-19 Diagnosis Based on Non-Invasive Measurements

Maghdid et al. [70] planned for the invisible COVID-19 diagnostic system based on smart phone sensors. Smart phones may be used in the proposed system to gather possible patient disease features. For example, through a recording feature, sensors can detect a patient’s voice and detect the body temperature of a patient via a finger recognition function. The data collected are subsequently transferred to a cloud server funded by AI for disease diagnosis and analysis. Indeed, it takes quite a while to compare different CT pictures, and radiologists cannpt physically finish their comprehension. In this way, the new system allows for radiologists and empowers individuals in suspicious cases to make successful and accurate decisions.

## 3. ML and DL for Drug and Vaccine Development

In combination with a large amount of data, the capacity for automatic abstract component learning has had a major impact on the efficacious use of ML. Drug discovery and vaccination affected two fields of high importance, where ML provided integrated property predictions, behaviour prediction, reaction prediction, and ligand-protein interactions. mDiverse drug development programs and vaccines for SARS-CoV-2 and COVID-19 have been suggested to focus on proteomics and genomics studies. One of the main contributions to intelligent medicine is the use of ML and DL in the development of new medicines and vaccines, and it plays a major function in the battle against COVID-19.

### 3.1. ML and DL For Vaccine Development

In this context, ML and DL play two important supporting roles: the dissemination of vaccine components by observing the viral protein structure, and helping medical researchers to review a large number of important research papers at an incredible rate. Three main types of vaccines are available: vaccines for any pathogen, such as flu or MMR, use deadly or compromised immune system infections; subunit vaccines (e.g., pertussis, shingles) only use part of the virus, such as protein; and, vaccines for nucleic acid inject viral genes into human cells to improve the response of the body [71]. The newest is the COVID-19 vaccine, which began trials in the United States this week. AI helps to speed up the growth of subunits and nucleic acids.

In analysing how it functions, understanding protein composition is critical. Researchers can produce drugs that work in various protein shapes once the situation is acknowledged. However, testing any protein structure will take quite a long time before finding its unique 3D structure. The method of evaluating protein structure and its genetic sequence can be simplified by AI systems that are based on DL.

Google DeepMind [72,73] introduced AlphaFold in January, an advanced and specialised system that forecasts the formation of 3D proteins while using their genetic sequence. In the beginning of March, the system was tested in COVID-19. In order to asses the scientific community for understanding the virus, DeepMind has published a protein prediction for several untreated proteins that are related to SARS-CoV-2, since it is the main cause for COVID-19. Meanwhile, scientists at the University of Texas at Austin and the National Institutes of Health have applied a standard biological method to establish the first 3D atomic map on the scale of a spike protein component of a virus that binds to human cells. Other Coronaviruses, including SARS-CoV and MERS-CoV, have spent years collaborating with the team that is responsible for this crucial breakthrough. Accurate predictions for this spike structure were provided by one of the predictions released by AlphaFold. Computer simulations for building 3D atom models on the SARS-CoV-2 protein scale neatly related to the results that were found on the UT Austin board were also used by [74] at the Institute for Protein Design at the University of Washington. By constructing new proteins to minimize Coronavirus, they are currently continuing on this work. In principle, these proteins will conform to a protein spike that protects healthy cells from being invaded by viral particles.

**Figure 6:**
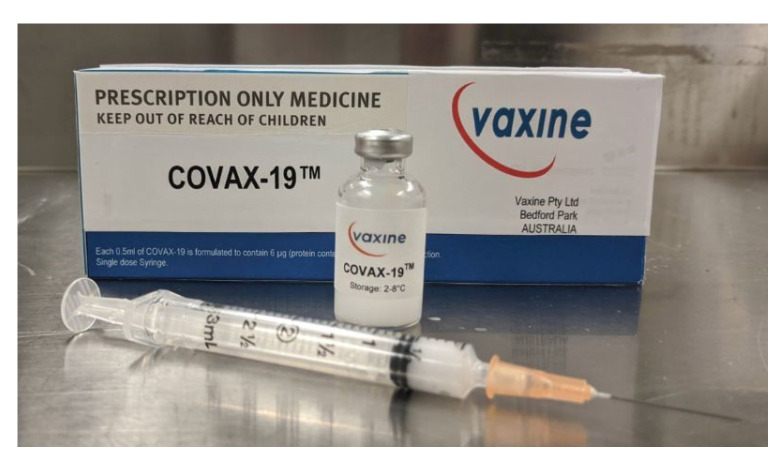
Covax-19™ is an Australian-designed COVID-19 vaccine modeled by AI-based technologies [76].

Researchers merged AI with cloud computing to stop the Spike protein from binding to the ACE2 receptor in human cells and to produce a possible vaccine for COVID-19 [75]. Flinders University researchers studied the COVID-19 virus and then applied their data to model a vaccine, called Covax-19, as shown in Figure 6.

In order to determine how the virus was harming human cells, the researchers utilized computer generated models of the S protein and its human receptor, the enzyme-converting angiotensin 2 (ACE2). Subsequently, they attempted to produce a vaccine that could prevent this mechanism. The team used the most innovative AI and advanced cloud computing technology to speed up the production of vaccines [73,75,77].

Herst et al. [78], detected GenBank’s SARS-CoV-2 protein sequence and applied MSA algorithm to specify the sequence of nucleocapsid phosphoprotein in future peptide sequencing. It also indicates that in EBOV (West African Epidemic 2013–2016) survivors, a peptide vaccine that is dependent on CD8 + T-cell immunity, is appropriate and feasible. Ong et al. [79] used the methods of ML and reverse vaccinology (RV) to forecast and test possible COVID-19 vaccines. In order to describe promising baptismal candidates, they used RV to analyse bioinformatics pathogen genomes. The SARS-CoV-2 sequence was discovered. The known proteins of the coronavirus types, including SARS-CoV, MERS-CoV, HCoV-229E, HCoVOC43, HCoV-NL63, and HCoV-HKU1, were obtained from Uniprot proteomes. They subsequently used Vaxign and Vaxign-ML to analyse and forecast the functioning of biological signals of the complete proteome of Coronaviruses. Next, using structural, vector support, proximity neighbor, random forest, and overgrowth (XGB) strategies, they formed the Vaxign-ML model in the context of ML and RV, and determined the level of the protein. In order to determine the rate of protegenicity of all WSH-CoV-2 proteins separating Wuhan-Hu-1, downloaded from NCBI, the most powerful XGB model was chosen. A good immunodeficiency vaccine is considered to be a protein with a protegenicity point that is greater than 90% (weight of F1-points >94% in five-fold combined validation). For phylogenetic study, the NSP3 protein was chosen and the immunogenicity of NSP3 was assessed by predicting MHC-I and MHC-II T cells and epitopes of Linear B cells.

Some other researchers [80] from the Computer Science and AI Laboratory (CSAIL) of MIT have currently adopted a novel strategy, in which they have applied an optimized ML-based method that choose peptides (short amino acid fibers) that are expected to give large vaccine numbers. The ”OptiVax” design software incorporates ways of designing new peptide medicines, evaluating existing vaccines and increasing the composition of existing vaccines. For their ability to express themselves in response to antibodies, peptides gain mechanical learning points in this program and they are chosen to expand coverage of those who can benefit from this vaccine.

Rahman et al. [81] applied immunoinformatics methods and relative methods in order to produce a SARS-CoV-2 anti-peptide vaccine consisting of S (spike), E (envelop), and M (membrane epitopes) protein. In order to predict B-specific epitopes in S-proteins, they applied the Ellipro antibody epitope predictive system. In order to forecast and visualize a specific protein sequence or B-cell epitope in a structure, Ellipro uses several ML techniques. In addition, the epitope-based vaccine design of COVID-19 was studied by Sarkar et al. [82]. They applied an SVM technique to forecast the toxicity of chosen epitopes. Prachar et al. [83] applied 19 joined epitope-HLA tools, including the IEDB, ANN, and PSSM algorithm, to forecast and validate 174 epitopes of SARS-CoV-2.

### 3.2. Drug Development

With the development of a predictive learning model, AI systems that are based on ML and DL are used in field design and they perform a fast-paced test to accurately represent the performance. The AI systems can easily classify drugs that can battle infectious diseases, such as COVID-19, with a drug recovery process. With this breakthrough, which is a medical evidence-based tool, will potentially enhance drug accessibility, preparation, care, and recorded patient outcomes COVID-19. Drug development is a very dangerous, lengthy, and costly phase. While it takes ten to fifteen years to make a new molecular venture, the success rate is only 2.01%, as reported by the Eastern Research Group (ERG) [84]. For the treatment of a never-considered medicinal indication, the idea of drug repurposing reuses old medications. It is an experimental strategy for the detection of pre-approved, discontinued, shelved, and investigational drugs for the treatment of other diseases with approved restatement. The development of conventional drugs generally involves five steps [85]:

- Drug discovery and development.
- Drug pre-clinical research.
- Drug clinical research.
- FDA drug review.
- FDA drug after-market safety control and development.

But only drug repurposing requires four steps [85]:

- Compound identification.
- Compound acquisition.
- Drug clinical research.
- FDA drug after-market safety control and development.

The discovery and development of new molecular structure are slow, time consuming, and expensive. Therefore, the best option is to reconstruct the approved drugs for SARS-COV-2 therapy. In this case, in the treatment of viral infection, Chloroquine (CQ) and its hydroxyl analogue Hydroxychloroquine (HCQ) have been identified. These drugs have antimalarial efficacy and they have also been shown to treat COVID-19 in vitro [86].

One AI-based drug development team focuses on the discovery at the molecular level of new drug-like compounds. Beck et al. [87] suggested a DL-based drug-target interaction model, called Molecule Transformer-Drug Target Interaction (MT-DTI), to predict potential drugs for COVID-19. SMILES strings and amino-acid sequences were used in the MT-DTI model in order to classify target proteins with 3D crystal structures. The findings showed that atazanavir, an antiretroviral drug that is used to treat and prevent the human immunodeficiency virus (HIV), is the best chemical compound with an inhibitory potency with Kd of 94.94 nM against 3C-like SARS-CoV-2 proteinase, followed by remdesivir (113.13 nM), efavirenz (199.17 nM), ritonavir (204.05 nM), and dolutegravir (336.91 nM). From the databases of NCBI, Drug Target Popular (DTC), and BindingDB, the authors obtained the amino acid sequences of 3C-like proteases and associated antiviral drugs and drug targets. In addition, they utilised a molecular docking and virtual screening method (AutoDock Vina) in order to determine the binding affinity between 3410 drugs and SARS-CoV-2 3CLpro. Six possible medicines, such as Remdesivir, Atazanavir, Efavirenz, Ritonavir, Dolutegravir, Kaletra (lopinavir/ritonavir), produced experimental results. Note that, in a clinical trial, Remdesivir looks promising.

Using effective machine learning techniques, two similarity-based methods, KronRLS [88] and SimBoost [89], have been proposed. However, there are two downsides to this matrix. First, the representation of features is reduced, which would cause the prediction faulty. Second, it involves the estimation of the matrix of similarity, which, in the training phase, will limit the maximum number of molecules. A deep learning based DTI model, DeepDTA [90], was proposed in order to address these limitations. It is a CNN-based end-to-end model that waives the feature engineering requirement. From a raw molecule and protein sequence, the model automatically finds useful features. The performance was seen on two publicly accessible DTI benchmarks, i.e., Sim-Boost and KronRLS. In order to extract features representations from the raw protein sequences and SMILES strings and train them, they used CNN blocks and combined these features representations to input into a deep CNN, naming it DeepDTA. They applied Smith–Waterman (S-W) and Pubchem Similarity algorithms to process the proteins and ligands’ pair-wise similarities, respectively. In tarining the patterns of the data, three alternative combinations applied this knowledge as input to the proposed ad enhanced DeepDTA model. The following are the three alternative combinations for training this model:

- Training only compound representation.
- Training only protein sequence representation.
- Training both protein representation and compound representations.

The last strategy is the combined model. The combined model is used in many researches [87,91,92] for COVID-19 drug repurposing.

They introduced a network-based DL method, called deepDR [93], for in silico drug repurposing by ten integrated networks. In particular, with the support of a deep Autoencoder, the deepDR understands medication high-stage features from the heterogeneous networks. Subsequently, with clinically recorded drug-disease pairs, the learned low-dimensional features representations of drugs is encoded and decoded together by a Variational Autoencoder to deduce applicants for licensed drugs that they were no longer initially allowed. DeepDR revealed a high performance of 90.8% AUC, surpassing other methods that are based on CNN and ML. Some of the other drug testing, repurposing, and discovery strategies that are based on deep learning for COVID-19 are DeepPurpose [91], kGCN [94], DeepChem [95], and D3Targets-2019-nCoV [96].

Bung et al. [97] undertook the progress of modern SARS-CoV-2 3CLpro chemical frameworks focused on deep learning technology. For protease inhibitor molecule molecules, they established an RL-based RNN model and found a tiny set that prefers chemical space. Eventually, 2515 SMILES format protease inhibitor molecules were collected from the ChEMBL database to train, in which each sequence of the SMILES was treated as a time series and as well as the location and symbol. The release of tiny molecules was embedded with minimal force into the 3CLpro structure and it was determined on the basis of a visual test note that was acquired by the selection of participants with anti-SARS-CoV-2. Tang et al. [98] analysed 3CLpro with a 3D structure that is similar to SARS-CoV and tested it as an appealing goal for the development of anti-COVID-19 drugs. They developed a comprehensive Q-learning network (called ADQN-FBDD) to produce leading SARS-CoV-2 3CLpro compounds. They collected 284 molecules as SARS-CoV-2 3CLpro inhibitors. These molecules are classified while using an enhanced BRICS technique in order to gain a SARS-CoV-2 3CLpro targeted library. Thereafter, the proposed ADQN-FBDD network trains each piece of the target and classifies the corresponding molecules with the leading elements. Through the Structure-Based Optimization Policy (SBOP), 47 alternatives were identified to the inhibitory effects on SARS-CoV-2 3CLpro in these compounds, which are considered to be anti-SARS-CoV-2 drugs. Table 4 shows a summary of drug development of COVID-19 based on AI-based ML and DL methods, providing a description of COVID-19 drug production that is based on AI-based ML and DL methods.

## 4. Challenges and Future Directions

In this section, we provide details on the challenges while using ML and DL to tackle the problem of COVID-19. Additionally, we show future research directions that ML and DL can contribute to the battle of COVID-19.

### 4.1. Challenges

AI-based ML and DL applications in COVID-19 research are currently facing several obstacles, such as legislation, scarcity, and unavailability of large-scale training data, vast noisy data and rumors, limited awareness of the intersection between computer science and medicine, data privacy and security issue, unreliable usability of text data, and more.

- **Regulation.** As the pandemic increases and maximizes the regular number of confirmed infected and deceased cases, numerous steps have been considered to contain this pandemic, e.g., Lockdown and social distancing. At the time of pandemic, authorities play a key part in identifying regulations and policies that can promote the participation of citizens, researchers, scientists, business owners, medical centers, technology giants, and major corporations to avert any barrier to COVID-19 prevention.
- **Scarcity and unavailability of large-scale training data.** Many AI-based DL techniques depend on large-scale training data, including medical imaging and different details of the environment. However, there are inadequate datasets available for AI, because of the rapid explosion of COVID-19. In reality, interpreting training samples is time-consuming and it may need trained medical professionals.
- **Noisy data and online rumors.** The challenges emerge from depending on portable online social media; without any significant changes, large audio information and false reports about COVID-19 have been reported in different online outlets. However, in judging and filtering audio and error data, AI-based ML and DL algorithms appear to be slow. Additionally, with the usage of noisy data, the results of the AI-based ML and DL algorithms become biased. The usage, functionality, and performance of AI-based methods are reduced by this issue, particularly in pandemic predictions and spread analysis.
- **Lacking in the intersection of computer science and medicine fields.** Most AI-based ML and DL researchers come from computer science major, but strong specialization in medical imaging, bioinformatics, virology, and many other related fields is also required to involve other medical knowledge for the use of ML and DL in the COVID-19 war. To deal with COVID-19, it is therefore important to organize the collaboration of experts from various fields and incorporate the information of multiple studies.
- **Data privacy and protection.** The cost of collecting personal privacy data in the age of big data and AI is very weak. Many governments aim to collect a range of personal information, including ID number, contact number, personal data, and medical data, in the face of public health issues, such as COVID-19. A problem worth addressing is how to efficiently preserve your privacy and human rights throughout AI-based discovery and processing.
- **Incorrect structural and unstructural data (e.g., image, text, and numerical data).** Having an ambiguous and incorrect information in text descriptions can be a challenge. Huge quantities of information from different sources can be incorrect. Additionally, excess data makes it impossible for valuable pieces of information to be extracted.
- **COVID-19 early diagnosis using medical imaging e.g., Chest X-ray and CT scan.** Dealing with unbalanced datasets due to inadequate medical imaging and long training period knowledge from COVID-19 and unable to clarify the challenges of the findings.
- **Screen and triage patients, find functional therapies and cures, risk assessments, survival predictions, health care, and medical resource planning.** The challenge is to collect physical attributes and treatment results for patients. Another difficulty is to deal with poor quality data that can lead to forecasts that are biased and unreliable.

### 4.2. Future Research Direction

AI-based ML and DL systems can also take part to the war against COVID-19 from the next possible directions.

- **Detection of non-contact disease.** The use of automated image classification in X-ray and CT imaging will effectively prevent the chance of disease transmissions from patients among radiologists during COVID-19 epidemics. For patient pose, the detection of X-ray and CT images, and camera facilities, AI-based ML and DL systems can be involved.
- **Remote video diagnosis and consultations.** It is possible to use the combination of AI and Natural Language Processing (NLP) techniques to build remote video diagnostic programs and robot systems in order to provide COVID-19 patient visits and first group diagnoses.
- **Biological research.** AI-based ML and DL systems can be used in the context of biological research to identify protein composition and viral factors via accurate biomedical knowledge analysis, such as significant protein structures, genetic sequences, and viral trajectories.
- **Drug development and vaccination.** AI-based ML and DL systems can not only be applied to identify possible drugs and vaccines, but they can also be used to mimic drug-protein and vaccine-receptor interactions, thus predicting future drug and vaccine reactions for people with various COVID-19 patients.
- **Identification and screening of fake information.** AI-based ML and DL systems can be applied to minimize and delete false news and audio data on internet forums in order to provide credible, factual, and scientific information about the COVID-19 outbreak.
- **Impact assessment and evaluation.** In order to analyse the effect of various social control modes on disease transmission, different types of simulations may use AI-based ML and DL systems. They can then be used to assess reliable and scientific approaches for disease control and prevention in the population.
- **Patient contact tracking.** AI-based ML and DL system can monitor and track the characteristics of people neighboring to patients with COVID-19 by creating social networks and knowledge graphs, thereby accurately predicting and monitoring the potential spread of the disease.
- **Smart robots.** In programs, such as sanitation in public areas, product delivery, and patient treatment without the need for human resources, intelligent robots are supposed to be used. This will stop the spreading of the virus from COVID-19.
- **Future work with descriptive AI-based ML and DL techniques.** The efficacy of deep learning models and graphical characteristics that lead to the differences between COVID-19 and other forms of pneumonia needs to be clarified. This will help radiologists and practitioners to gain an awareness of the virus and accurately analyse the possible X-ray and CT scans of the Coronavirus.
- **COVID-19 diagnosis and treatment, which one is important?** Both are essential, but finding a cure for COVID-19 is definitely more essential. We find that most of the existing AI-based ML and DL strategies are more centered on identifying COVID-19 from the existing literature in this survey. In order to find the COVID-19 treatment, more future research work based on ML and DL is required.

## 5. Final Comments

The COVID-19 outbreak has had a profound effect on the wellbeing of people world wide and the number of disease-related deaths continues to grow globally. Although technology has penetrated our daily lives with great success, especially in ML and DL, AI has also contributed to supporting people in the difficult battle against COVID-19. DL is just one possible successful way to provide promising data driven solutions to help the humanity to handle with COVID-19.

In this survey, we explored the AI-based ML and DL methods for COVID-19 diagnosis and treatment. In addition, in the war against COVID-19, we summarized the AI-based ML and DL methods and the available datasets, tools, and performance. This survey offers a detailed overview of the existing state-of-the-art methodologies and applications for ML and DL researchers and the wider health community with descriptions of how ML and DL and data will boost the status of COVID-19, and more studies for avoiding the outbreak of COVID-19. Challenges and potential guidance were also presented whileusing ML and DL.

## Figures and Tables

**Table 1:** A summary of predictive performance of Artificial Intelligence (AI)-based ML and DL techniques for diagnosing COVID-19 using chest X-ray and CT images.

Author	Data	ML/DL Method	Accuracy	AUC	Sensitivity	Specificity
**Li et al. [28]**	A total of 4356 CT images from 3322 patients obtained from six clinics (1296 images for COVID-19, 1735 for CAP and 1325 for non-pneumonia).	COVNet using pre-traind ResNet-50	N/A	96% AUC	87%	92%
**Butt et al. [29]**	A total of 618 CT images (219 images from 110 COVID-19 patients, 224 CT images from 224 patients with influenza-A viral pneumonia, and 175 CT images from healthy people).	Location-attention network and ResNet-18	86.7%	N/A	N/A	N/A
**Ghoshal and Tucker [30]**	A total of 5941 CT images for four classes (1583 normal, 2786 bacterial pneumonia, 1504 viral pneumonia, and 68 COVID-19)	Drop-weights based Bayesian CNNs	89.92%	N/A	N/A	N/A
**Bai et al. [31]**	Unknown number of chest CT images collected from 133 COVID-19 patients of which 54 patients had severe/critical cases.	MLP and LSTM	N/A	95%	95%	95%
**Jin et al. [32]**	496 chest CT scans from COVID-19 patients and 1385 non-COVID-19 CT scans.	CNN	N/A	97.17%	90.19%	95.76%
**Jin et al. [33]**	A total of 1136 CT images (723 COVID-19 confirmed COVID-19 cases from five hospitals).	3D UNet++ and ResNet-50	N/A	N/A	97%	92%
**Narin et al. [34]**	Only 50 COVID-19 chest X-ray scans and 50 for non-COVID-19.	Pre-trained ResNet-50	98%	N/A	N/A	N/A
**Wang and Wong [35]**	16,756 chest X-ray scans from 13,645 COVID-19 patients.	COVID-Net	92.4%	N/A	91.0%	N/A
**Gozes et al. [36]**	Unknown number of CT scans collected from 157 Chinese and U.S. COVID-19 patients.	ResNet-50	N/A	97%	98.2%	92.2%
**Chowdhury et al. [37]**	A total of 3487 chest CT scan images (1579 normal, 1485 viral pneumonia, and 423 COVID-19.	AlexNet, ResNet-18, DenseNet201, and SqueezeNet	99.7%	N/A	99.7%	99.5%
**Maghdid et al. [38]**	A total of 170 COVID-19 X-ray scans and a total of 361 COVID-19 CT scans of collected from five different places.	CNN and pre-trained AlexNet models	98% on X-ray scans and 94.1% on CT scans	N/A	100% on X-ray scans and 90% on CT scans	N/A
**Apostolopoulos and Mpesiana [39]**	A total of 1428 chest X-ray scans (224 COVID-19, 700 pneumonia patient, and 504 normal).	Pre-trained VGG-19	93.48%	N/A	92.85%	98.75%
**Sethy and Behera [40]**	Chest X-ray scans of 25 COVID-19 positive and 25 negative.	ResNet-50 and SVM	93.28%	N/A	97%	93%
**Hemdan et al. [41]**	Chest X-ray scans 25 COVID-19 positive and 25 Normal patient.	COVIDX-Net	90%	N/A	N/A	N/A
**Wang et al. [6]**	Chest CT scans of 195 COVID-19 positive and 258 COVID-19 negative patients.	M-Inception	89.5%	N/A	87%	88%
**Zheng et al. [42]**	Chest CT images of 313 COVID-19 and 229 non-COVID-19 patients.	U-Net and a 3D Deep Network	90%	N/A	95%	95%
**Hall et al. [43]**	A total of 455 chest X-ray (135 of COVID-19 and 320 of viral and bacterial pneumonia.	Pre-trained ResNet-50	89.2%	95%	N/A	N/A
**Ni et al. [44]**	CT images of 96 COVID-19 positive collected from three hospitals in China.	Deep CNN	73%	N/A	95%	95%
**Apostolopoulos et al. [45]**	A total of 3905 X-ray scans for several classes of disease including COVID-19.	MobileNet-v2	99.18%	N/A	97.36%	99.42%
**Shi et al. [46]**	CT images of 2685 collected from three hospitals, 1658 of COVID-19 positive cases and 1027 cases were CAP patients.	RF	87.9%	N/A	90.7%	83.3%
**Loey et al. [26]**	Chest X-ray scans of 307 for COVID-19, normal, pneumonia, bacterial, and pneumonia virus.	GAN with AlexNet, GoogLeNet, and ResNet18 pre-trained models.	80.56%	N/A	N/A	N/A
**Maia et al. [47]**	A total of 437 X-ray images (217 COVID-19, 108 other diseases, and 112 healthy).	Different Convolutional Support Vector Machines (CSVMs)	98.14%	N/A	N/A	N/A

**Table 2:** COVID-19 severity stages using a score-based system.

Parameters	Severity Score				
	0	1	2	3	4
Extent of Lung Involvement	No	<25%	25–50%	50–75%	>75%
Degree of ambiguity	No	Ground glass ambiguity	Unification	White-out	-

**Table 3:** An overview of AI-based ML and DL methods for COVID-19 speech and audio analysis.

Author	Usage	ML/DL Method	Dataset	Predictive Performance
**Miranda et al. [63]**	TB Diagnosis	MFB, MFCC, STFT with CNN	Google audio dataset which includes 1.8 million Youtube Freesound videos and audio database	94.6% AUC
**Yadav et al. [64]**	Asthmatic Discovery	INTERSPEECH 2013 (The basis of the Computational Paralinguistics challenge acoustic features)	Speech from 47 asthmatic and 48 healthy controls	48% Accuracy
**Larson et al. [65]**	Avoid recording of speech while hearing cough	PCA on audio spectrograms, FFT coefficients and random separation	Acted cough from 17 patients having 8 people cough due to cold weather, 3 patients because of Asthama, 1 patient because of allergies and 5 patients who cough due to chronic cough condition	92% of True positive rate and 50% of false positive rate
**Simply et al. [66]**	Obstructive sleep apnea (OSA) acquisition from sounds breathing in speech	MFCCs have a single layer neural network for respiration and MFCCs, strength, tone, kurtosis and ZCR with OV SVM separation	90 Male subjects’ speech and sleep quality measures using WatchPAT	50% for breathing detection using Cohen’s kappa coefficient and 54% using OSA detection
**Routray et al. [67]**	Automatic speech breathing average rating	Cepstrogram and support Vector machine with radial base work	16 recording of speeches 21-year-old group participants age means	89% F1-measure
**Nallanthighal and Strik [68]**	Receiving a breathing signal in conversational speech	Pectrogram with CNN and RNN	20 healthy speeches recordings using a microphone and a breathing signal using two respirator transducers belts	91.2% respiratory sensitivity
**Partila et al. [69]**	Pressure detection in speech in cases such as a car accident, domestic violence, and conditions near death	LLD and functional characteristics are obtained using openSMILE with SVM and CNN separators	312 emergency call recording of 112 Emergency Cable Emergency Plan from the Czech Republic	87.9% accuracy using SVM and 87.5% Accuracy using CNN

**Table 4:** A summary of drug development of COVID-19 based on AI-based ML and DL methods [99].

Author	Data	Method	Role of ML/DL Method	COVID-19 Target	Potent In Drugs
**Moskal et al. [100]**	A random subset of 6000 small molecules from the ZINC database and divided it randomly into the training and test sets in 5:1 proportion	VAE, CNN, LSTM, and MLP	Help in generating SMILES strings and molecules	N/A	110 Drugs
**Beck et al. [87]**	Drug Target Common (DTC) and BindingDB datasets	MT-DTI	Predict binding affinity between drugs and protein targets	3CLpro, RdRp, Helicase	6 Drugs
**Hu et al. [101]**	A dataset contains 4895 commercially drugs	DL	Predict binding between drugs and protein targets	3CLpro, RdRp	10 drugs
**Kadioglu et al. [102]**	FDA-approved drugs for drug repositioning, Natural compound dataset from literature mining, and ZINC dataset to select compounds interacting with SARS-CoV-2 target proteins.	NN and Naive Bayes	Construct drug likelihood prediction model	spike protein and more	3 drugs
**Hofmarcher et al. [103]**	Binary ECFP4 fingerprints folded to a length of 1024 and 75 output units of ChemAI	ChemAI	Classify the moecules effects on COVID-19 proteases	3CLpro, PLpro	30,000 molecules
**Bung et al. [97]**	A dataset of 1.6 million drug-like small molecules from the ChEMBL database	RNN and RL	Classify protease inhibitor molecules	3CLpro	31 compounds
**Zhavoronkov et al. [104]**	Crystal structure of 2019-nCoV 3C-like protease	28 ML	Generate new molecular structures for 3CLpro	3CLpro	100 molecules
**Tang et al. [98]**	SARS-CoV 3CLpro inhibitors (284 molecules)	RL and DQN	Predict molecules and lead compounds for each target fragment	3CLpro	47 compounds

## Data Availability

This study did not report any data.
